# Using Clinician-Patient WeChat Group Communication Data to Identify Symptom Burdens in Patients With Uterine Fibroids Under Focused Ultrasound Ablation Surgery Treatment: Qualitative Study

**DOI:** 10.2196/43995

**Published:** 2023-09-01

**Authors:** Jiayuan Zhang, Wei Xu, Cheng Lei, Yang Pu, Yubo Zhang, Jingyu Zhang, Hongfan Yu, Xueyao Su, Yanyan Huang, Ruoyan Gong, Lijun Zhang, Qiuling Shi

**Affiliations:** 1 State Key Laboratory of Ultrasound in Medicine and Engineering College of Biomedical Engineering Chongqing Medical University Chongqing China; 2 School of Public Health Chongqing Medical University Chongqing China

**Keywords:** social media, group chats, text mining, free texts, symptom burdens, WeChat, natural language processing, NLP

## Abstract

**Background:**

Unlike research project–based health data collection (questionnaires and interviews), social media platforms allow patients to freely discuss their health status and obtain peer support. Previous literature has pointed out that both public and private social platforms can serve as data sources for analysis.

**Objective:**

This study aimed to use natural language processing (NLP) techniques to identify concerns regarding the postoperative quality of life and symptom burdens in patients with uterine fibroids after focused ultrasound ablation surgery.

**Methods:**

Screenshots taken from clinician-patient WeChat groups were converted into free texts using image text recognition technology and used as the research object of this study. From 408 patients diagnosed with uterine fibroids in Chongqing Haifu Hospital between 2010 and 2020, we searched for symptom burdens in over 900,000 words of WeChat group chats. We first built a corpus of symptoms by manually coding 30% of the WeChat texts and then used regular expressions in Python to crawl symptom information from the remaining texts based on this corpus. We compared the results with a manual review (gold standard) of the same records. Finally, we analyzed the relationship between the population baseline data and conceptual symptoms; quantitative and qualitative results were examined.

**Results:**

A total of 408 patients with uterine fibroids were included in the study; 190,000 words of free text were obtained after data cleaning. The mean age of the patients was 39.94 (SD 6.81) years, and their mean BMI was 22.18 (SD 2.78) kg/m^2^. The median reporting times of the 7 major symptoms were 21, 26, 57, 2, 18, 30, and 49 days. Logistic regression models identified preoperative menstrual duration (odds ratio [OR] 1.14, 95% CI 5.86-6.37; *P*=.009), age of menophania (OR –1.02 , 95% CI 11.96-13.47; *P*=.03), and the number (OR 2.34, 95% CI 1.45-1.83; *P*=.04) and size of fibroids (OR 0.12, 95% CI 2.43-3.51; *P*=.04) as significant risk factors for postoperative symptoms.

**Conclusions:**

Unstructured free texts from social media platforms extracted by NLP technology can be used for analysis. By extracting the conceptual information about patients’ health-related quality of life, we can adopt personalized treatment for patients at different stages of recovery to improve their quality of life. Python-based text mining of free-text data can accurately extract symptom burden and save considerable time compared to manual review, maximizing the utility of the extant information in population-based electronic health records for comparative effectiveness research.

## Introduction

Uterine fibroids (UFs) are the most common benign diseases of the reproductive tract in women of reproductive age, and their prevalence has been increasing in recent decades, ranging from 50% to 77% [1]. UFs are commonly observed in women of childbearing age, widowhood, and uncoordinated sexual life [2]. They can cause abdominal masses, increased menstrual flow, dizziness, fatigue, frequent urination, and secondary dysmenorrhea. Therefore, alleviating adverse symptoms and improving the quality of life are the primary purposes of UF treatment.

Focused ultrasound ablation surgery (FUAS) is an emerging technology for the treatment of UFs without any visible incision [3-6]. The median hospitalization time for patients is 2 days [7]. Postoperative complications may occur from hospitalization to several months after surgery, and a substantial proportion of patients may experience symptoms related to disease recurrence. Frequent monitoring using standardized patient-reported outcome (PRO) tools could improve patients’ quality of life in both open and minimally invasive surgical practice [8]. Whether this approach works similarly in noninvasive surgery settings is still unknown because of the lack of understanding of what patients experience throughout recovery and disease recurrence.

Unlike those with malignant diseases, patients with UFs are not rushed to seek professional medical advice when a symptom bothers them, and they tend to turn to the internet for help [9]. Information from Facebook or Twitter [1-4] has served as a source for identifying patients’ experiences and health care needs during the perioperative phase. In China, the most widely used social media platform is WeChat, covering approximately 1.2 billion internet users of all ages [10]. Clinician-patient WeChat groups are popular in all types of hospitals and are an effective instrument for patient care after surgery. Unlike discussion forums open to all viewers on other social media, clinician-patient WeChat groups are established by medical staff, and since only patients treated by those medical staff are granted access, the groups are relatively private. In these groups, patients may report their symptoms, consult with doctors for their medical examination reports, and seek guidance for their next clinical visits. Medical staff respond within 24 hours, with explanations or suggestions. A patient may share their experience with anything related to their diseases and treatments with other patients because they are grouped for similar reasons. Because of insufficient medical personnel resources and heavy medical workloads, clinician-patient communication in China is extremely brief in clinics, and patients might feel that their doctors do not care about them. Thus, clinician-patient WeChat groups have been widely accepted as a complementary approach to relieve tension between doctors and patients [5].

However, the use of information from clinician-patient WeChat groups has been limited. As a nonprofessional communication platform, in addition to disease-related text, the WeChat group chat is also filled with a large amount of noisy data, which interferes with the artificial intelligence–based learning of natural language processing (NLP) [11], causing inaccurate data extraction of disease- and treatment-related information. To appropriately use and integrate information from clinician-patient WeChat communication, specific algorithms for this type of natural language are necessary.

Therefore, with the availability of patients’ postdischarge experience from the clinician-patient WeChat groups and disease and treatment information from the electronic health records (EHRs), we aimed to develop an algorithm for using NLP techniques to extract post-FUAS symptoms experienced by patients from the clinician-patient WeChat group text, describe symptom burdens before and after FUAS for UFs, and determine the risk factors of postoperative symptom burdens [6-9].

## Methods

### Study Design

This study used a method that consisted of WeChat free-text NLP and the analysis of quantitative data from the EHR.

### Ethics Approval

The Institutional Review Board of Chongqing Haifu Hospital approved the protocol (approval number: 2020-003). Informed consent was waived for the retrospective design. The registration number of the China Clinical Trial Center was ChiCTR2200056735.

### Data Source

We used ABBYY FineReader 14 to extract the texts from the screenshots of clinician-patient WeChat groups, including the communication between patients and attending physicians from January 2010 to December 2020. We obtained screenshots of clinician-patient WeChat groups from the patient management project after FUAS treatment, which consisted of a continuous report from patients on their state and the clinician’s response to patients, as shown in Figure S1 in Multimedia Appendix 1.

### Data Cleaning

We filtered the WeChat group chat texts using the following keywords: “肌瘤” (“fibroid” in Chinese), “子宫肌瘤” (“uterine fibroid” in Chinese), “多发子宫肌瘤” (“multiple fibroids” in Chinese), “多肌” (“multiple muscle” in Chinese), and “子肌” (“uterine muscle” in Chinese). For text mining, we removed all the emojis and created a script to remove URLs and texts of hospital examination records. To ensure that the data only include the text related to the research topic, we cleaned the texts by removing words and characters that were of little or no analytical value (eg, “早上” [“morning” in Chinese] and “下午” [“afternoon” in Chinese]). Finally, only the communication texts of patients related to UFs were preserved [12-14]. The cleaned data were randomly divided into a training data set (30%) and a validation data set (70%).

### Manual Coding

We convened an expert panel of gynecologists, computer programmers, and medical informatics professionals, which categorized symptoms as present or absent. Symptoms were coded as present if they appeared in the WeChat group chat texts (eg, menorrhagia, dysmenorrhea, menstrual disorder, or prolonged menstrual period). In contrast, if the symptom appeared in the WeChat group chat texts but included a negation term, it was coded as absent (eg, no dysmenorrhea and no vaginal secretion).

Once symptoms were defined, 2 members of the study team (annotators) reviewed each note in the training data set. After the 2 annotators coded their notes, a third investigator reviewed the annotations and resolved discrepancies. Doccano software was used to annotate the documents. Doccano allows reviewers to view words with coding concordance (highlighted in green) or discordance (highlighted in red). If the first 2 annotators were unsure of how to resolve discordant coding, all 3 study team members resolved the coding through discussion and, when necessary, formalized the iterative coding rules. The frequency distribution of manually coded symptoms is shown in Figure S2 in Multimedia Appendix 1.

### Establishment of Symptomatic Corpus

Manual coding generated an initial corpus of relevant symptomatic key terms or phrases that we used to define the features in our models (Table S1 in Multimedia Appendix 1). Given that the Doccano tool uses regular expressions to define features in models, we wrote a series of regular expressions using the symptomatic key terms and phrases described above, shortened the list of regular expressions by counting the number of features detected in our training data set, and removed those that did not have any counts [15-18].

By manually coding 30% of the WeChat group chat texts, we obtained a corpus of symptomatic key terms and phrases. Subsequently, we developed a text-processing approach based on Python’s regular expression skills. The corpus returned from the manual coding was further processed using a number of string-processing functions that support regular expression–like features. We used these functions to process the WeChat group chat texts to identify the postoperative symptom patterns.

### Extraction of Identity Information

We used regular expressions in Python to extract patients’ identity information (patient ID and name) from the WeChat group chats. Duplicates were removed. We used these identities to match the patients in the EHR and extracted disease and treatment information for each patient obtained from the WeChat group chat text.

### Algorithm for Symptom Identification

We applied regular expression based on the corpus, which was attained after manual coding, and then identified the symptoms in the corpus (Table S1 in Multimedia Appendix 1) from the WeChat group chat texts. Using the parsing capability of regular expression in Python, we systematically searched for symptoms on uncoded WeChat group chat texts. After a symptom was identified with all different key terms or phrases, we moved to another symptom until key terms or phrases of the corpus have been browsed through; simultaneously, we pinpointed the time when each symptom occurred.

### Data Analysis

Symptoms were summarized as frequencies of presence before and after FUAS separately, and continuous variables were expressed as means and SDs or medians and IQRs. Categorical data were presented as percentages. The *t* test (2-tailed) and Wilcoxon paired rank-sum test were used to compare baseline data between the non-dysmenorrhea and dysmenorrhea groups for continuous variables as appropriate.

The text mining of WeChat group chats was compared with manual review findings (gold standard). The agreement was assessed with κ statistics. κ measures the proportion of agreement between methods after removing any chance agreement. For κ, values of 0.61 to 0.80 are considered good, and values of 0.81 to 1.00 are considered excellent.

We defined the top 10 symptoms presented before surgery as *disease-related symptoms* and symptoms that emerged in the text of WeChat after surgery as *treatment-related symptoms*. Multivariate generalized estimating equation (GEE) models were constructed to identify risk factors for the prevalence of symptoms over time (before FUAS and 1, 2, 3, and 4-6 months after FUAS). Factors included in GEE models were age (≤40 years vs >40 years), BMI (≤23.37 kg/m^2^ vs >23.37 kg/m^2^), pregnancy (≤2 vs >2), parity (≤1 vs >1), and abortion (≤2 vs >2). The effects of risk factors were presented as odds ratios (ORs) with 95% CIs. Using the Bonferroni correction for multiple comparisons of risk factor identification, the statistical significance level was set at the adjusted cutoff of *P* value = .05 / number of risk factors.

We used the GEE model to describe the trajectories of the 9 most common symptoms in the order of prevalence (vaginal secretion, dysmenorrhea, menstrual disorder, prolonged menstrual periods, lower abdominal pain, menstrual blood clots, menorrhagia, dizziness, and fatigue) over the 5 time points. Two piecewise (from before to 1 month after FUAS and from 1 month to 4-6 months after FUAS) random coefficient models were used to analyze trends of symptom presence before and after surgery. All *P* values were 2-tailed, and statistical significance was set at the conventional cutoff of *P*<.05. All data analyses were performed using the statistical software SAS (version 9.4; SAS Institute).

## Results

### Data Extraction

We captured the basic information of the patients’ medical records from 20 clinician-patient WeChat groups of 5 attending doctors. After excluding patients with other gynecological diseases, 408 patients with UFs were included in this analysis. A total of 8188 screenshots were obtained. We extracted 939,735 characters after image recognition and free-text cleaning. Finally, the group chat texts of patients with UFs contained 190,000 words. Five time points were extracted, including the preoperative period and 1, 2, 3, and 4-6 months after surgery.

### Demographics

Patient baseline characteristics are presented in Table 1. The mean age was 39.94 (SD 6.81; IQR 35-45) years; the mean BMI was 23.47 (SD 29.37; range 20.20-23.83) kg/m^2^; and the median number of pregnancies, abortions, Caesarean sections, and parity were 2 (IQR 1-3), 2 (IQR 1-3), 0 (IQR 0-1), and 1 (IQR 1-1), respectively. Preoperative symptoms were present in 59.1% (241/408) of patients.

**Table 1:** Demographic characteristics of the study population.

Demographic characteristics			Value (N=408)
Age (years), mean (SD; IQR)			39.94 (6.81; 35-45)
BMI (kg/m^2^; n=402), mean (SD; IQR)			23.47 (29.37; 20.20-23.83)
Pregnancy (n=406), mean (SD; IQR)			2.36 (1.87; 1-3)
Abortion (n=323), mean (SD; IQR)			1.90 (1.43; 1-3)
Caesarean section (n=290), mean (SD; IQR)			0.46 (0.60; 0-1)
Parity (n=326), mean (SD; IQR)			0.98 (0.63; 1-1)
Age of menarche (years; n=405), mean (SD; IQR)			13.29 (1.51; 12-14)
Menstrual period (days; n=407), mean (SD; IQR)			6.13 (2.59; 5-7)
Menstrual cycle (days; n=405), mean (SD; IQR)			28.76 (4.07; 27-30)
UF^a^-LR^b^ size (mm; n=161), mean (SD; IQR)			53.8 (17.7; 45-61)
UF-FB^c^ size (mm; n=381), mean (SD; IQR)			52.7 (17.9; 40-63)
Length of UF (mm; n=399), mean (SD; IQR)			60.1 (22.0; 45-70)
Length of stay (days; n=400), mean (SD; IQR)			3.10 (1.97; 2-3)
**Readmission, n (%)**			
	Yes	44 (10.8)	
	No	364 (89.2)	
**Location of UF (n=387), n (%)**			
	Submucosal	22 (5.7)	
	Intramural	347 (89.7)	
	Subserosal	18 (4.6)	
**Employment status (n=407), n (%)**			
	Unemployed	167 (41)	
	Part-time employment	1 (0.2)	
	Full-time employment	239 (58.7)	
**Ethnicity (n=407), n (%)**			
	1	389 (95.6)	
	6	2 (0.5)	
	7	16 (3.9)	
**Number of UFs (n=404), n (%)**			
	Single	140 (34.6)	
	Multiple	264 (65.4)	
**Marriage status** **, n (%)**			
	1	321 (78.7)	
	2	34 (8.3)	
	3	1 (0.2)	
	5	50 (12.2)	
	6	2 (0.5)	
**Extent of dysmenorrhea** **, n (%)**			
	None	281 (68.9)	
	Mild	89 (21.8)	
	Moderate	29 (7.1)	
	Severe	9 (2.2)	

^a^UF: uterine fibroid.

^b^LR: left to right.

^c^FB: front to back.

### Corpus of UFs

According to the manual coding of the concept of postoperative symptoms, the most reported symptomatic concepts were menorrhagia, dysmenorrhea, prolonged menstrual periods, menstrual blood clots, dizziness, fatigue, lower abdominal pain, menstrual disorder, and vaginal secretion (eg, “increased secretion,” “watery secretion,” “bloody secretions,” “excreted secretions,” and “vaginal secretion”). Tables S2 and S3 in Multimedia Appendix 1 present the symptom normalization mapping [7-10].

We extracted the frequency of symptoms before and after FUAS (Multimedia Appendix 2). The presence rates were significantly reduced after surgery for several symptoms, including menorrhagia (137/408, 33.6% vs 17/408, 4.2%), dysmenorrhea (135/408, 33.1% vs 64/408, 15.7%), prolonged menstrual period (86/408, 21.1% vs 34/408, 8.3%), menstrual blood clots (55/408, 13.7% vs 28/408, 6.9%), dizziness (55/408, 13.7% vs 10/408, 2.5%), and fatigue (55/408, 13.7% vs 10/408, 2.5%; all *P*<.001). Two symptoms (vaginal secretion [0/408, 0% vs 64/408, 15.7%] and menstrual disorder [16/408, 3.9% vs 62/408, 15.2%]) were reported significantly more frequently after surgery than before FUAS (all *P*<.001). No statistical difference was found in lower abdominal pain before and after FUAS (40/408, 9.8% vs 33/408, 8.1%; *P*=.39; Table 2).

**Table 2:** Comparison of the prevalence of preoperative and postoperative symptoms (N=408).

Symptoms	Preoperative prevalence, n (%)	Postoperative prevalence, n (%)	*χ*^2^ (*df=1*)	*P* value
Vaginal secretion	0 (0)	64 (15.7)	69.447	<.001
Dysmenorrhea	135 (33.1)	64 (15.7)	33.502	<.001
Menstrual disorder	16 (3.9)	62 (15.2)	29.995	<.001
Prolonged menstrual periods	86 (21.1)	34 (8.3)	26.418	<.001
Lower abdominal pain	40 (9.8)	33 (8.1)	0.737	.39
Menorrhagia	137 (33.6)	17 (4.2)	115.259	<.001
Menstrual blood clots	55 (13.7)	28 (6.9)	10.404	<.001
Dizziness	55 (13.7)	10 (2.5)	34.882	<.001
Fatigue	55 (13.7)	10 (2.5)	34.882	<.001

### Symptom Changing Over Time

The trajectories of 10 symptoms were analyzed. Menorrhagia, prolonged menstrual periods, menstrual blood clots, dizziness, lower abdominal pain, intensified dysmenorrhea, and frequent urination significantly decreased from the preoperative period to 4-6 months after FUAS treatment (all *P*<.001; Table 3).

**Table 3:** Generalized estimating equation model analysis of perioperative symptoms at 5 time points.

Variables	Estimate (time; SE)	*P* value
Menorrhagia	–1.4036 (0.1892)	<.001
Dysmenorrhea	–0.2571 (0.1492)	.08
Prolonged menstrual period	–3.7684 (0.9710)	<.001
Menstrual blood clots	–1.2824 (0.2552)	<.001
Dizziness	–1.8632 (0.4329)	<.001
Lower abdominal pain	–0.6222 (0.1309)	<.001
Menstrual disorder	0.0327 (0.0991)	.74
Vaginal secretion	–0.1359 (0.0754)	.07
Intensified dysmenorrhea	–2.3502 (0.7010)	<.001
Frequent urination	–2.1042 (0.4893)	<.001

**Figure 1:**
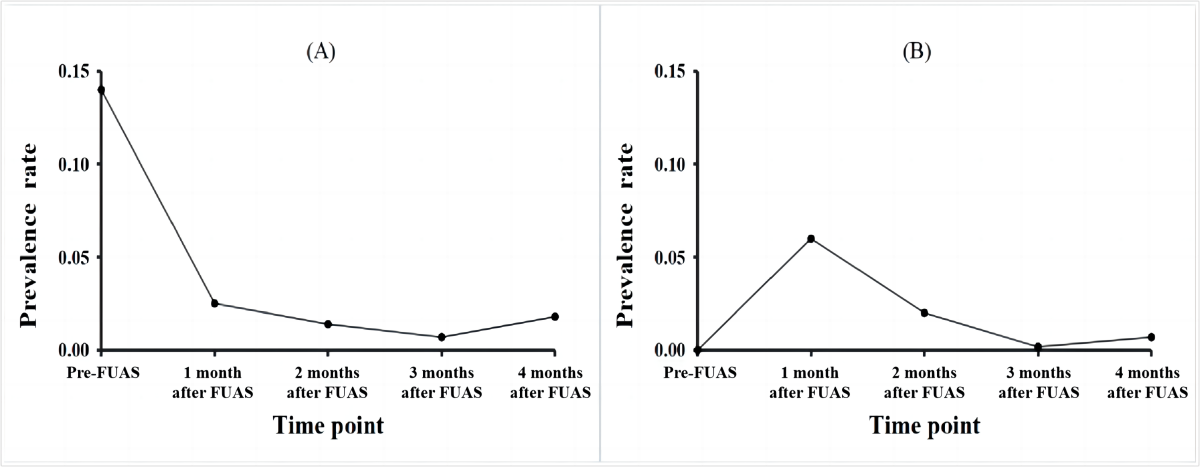
Two piecewise regression analysis for 2 symptoms during 5 time points in the perioperative period: (A) dysmenorrhea and (B) vaginal secretion. FUAS: focused ultrasound ablation surgery.

Two symptoms demonstrated significantly different trends of change over the 2 time period (from before to 1 month after FUAS and from 1 to 4 months after FUAS). The proportion of patients with dysmenorrhea tended to significantly decrease from the preoperative period to the first postoperative month (estimate –0.49; *P*=.03) and gradually stabilized after the first postoperative month (estimate 0.17; *P*=.16). The proportion of patients with vaginal secretion significantly increased from the preoperative period, peaking in the first postoperative month (estimate 1.43; *P*=.04), and decreased thereafter (estimate –1.08; *P*=.04; Figure 1).

### Association Between Preoperative Dysmenorrhea and Postoperative Symptoms

Compared to those without preoperative dysmenorrhea, patients with dysmenorrhea had a higher probability of reporting menstrual blood clots after FUAS (11.11% vs 4.76%, *P*=.02), whereas the differences in vaginal discharge, menorrhagia, prolonged menstrual period, advanced or delayed menstruation, dysmenorrhea, dizziness, lumbar and abdominal discomfort, and frequent urination were not significant (all *P*<.05; Table 4).

**Table 4:** Analysis of the difference between preoperative dysmenorrhea and postoperative symptoms.

Symptom and preoperative dysmenorrhea		Postoperative symptoms, n (%)	Statistics	*P* value
**Vaginal secretion**			–0.3169	.96
	Yes (n=135)	21 (15.6)		
	No (n=273)	43 (15.8)		
**Menstrual blood clots**			11.6663	.02
	Yes (n=135)	15 (11.1)		
	No (n=273)	13 (4.8)		
**Menorrhagia**			8.952	.12
	Yes (n=135)	21 (15.6)		
	No (n=273)	28 (10.3)		
**Prolonged menstrual period**				
	Yes (n=135)	9 (6.7)	–4.4473	.39
	No (n=273)	25 (9.2)		
**Menstrual disorder**			3.2851	.59
	Yes (n=135)	22 (16.3)		
	No (n=273)	38 (13.9)		
**Dysmenorrhea**			8.0487	.20
	Yes (n=135)	26 (19.3)		
	No (n=273)	39 (14.3)		
**Dizziness**			1.613	.64
	Yes (n=135)	4 (3)		
	No (n=273)	6 (2.2)		
**Lower abdominal pain**			8.1021	.12
	Yes (n=135)	15 (11.1)		
	No (n=273)	18 (6.6)		
**Frequent urination**			–0.8309	.73
	Yes (n=135)	1 (0.7)		
	No (n=273)	3 (0.01)		

### Factors Related to Symptoms

#### Preoperative Factors

As shown in Table 5, differences in age group (*P*=.006), BMI (*P*=.006), and the number of pregnancies (*P*=.04) between patients with and without dysmenorrhea were statistically significant in the patient population before FUAS. Patients aged ≤40 years (OR 0.55, 95% CI 0.36-0.84), those with a BMI greater than 24.0 kg/m^2^ (OR 1.88, 95% CI 1.20-2.94), or those with <2 pregnancies (OR 0.63, 95% CI 0.41-0.97) were more likely to have dysmenorrhea.

**Table 5:** Risk factor analysis for patients with preoperative dysmenorrhea.

Variables		Patients with dysmenorrhea (n=135), n (%)	Patients without dysmenorrhea (n=262), n (%)	*P* value	
**Age group (years)**					.006
	≤40	77 (57)	111 (42.4)		
	>40	58 (43)	151 (57.6)		
**BMI (kg/m^2^)**					.006
	<24.0	84 (62.2)	198 (75.6)		
	≥24.0	51 (37.8)	64 (24.4)		
**Pregnancy**					.04
	<2	60 (44.4)	88 (33.6)		
	≥2	75 (55.6)	174 (66.4)		
**Abortion**					.09
	<2	79 (58.5)	144 (55)		
	≥2	56 (41.5)	118 (45)		
**Caesarean section**					.35
	0	44 (32.6)	122 (46.6)		
	>0	37 (27.4)	78 (29.8)		
**Parity**					.75
	≤2	56 (41.5)	85 (32.4)		
	>1	79 (58.5)	177 (67.6)		
**Age of menophania**					.28
	<13	83 (61.5)	145 (55.3)		
	≥13	52 (38.5)	117 (44.7)		
**Menstrual period (days)**					.92
	<6	76 (56.3)	149 (56.9)		
	≥6	59 (43.7)	113 (43.1)		
**Menstrual cycle (days)**					.67
	<29	70 (51.9)	129 (49.2)		
	≥29	65 (48.1)	133 (50.8)		
**UF^a^-LR^b^ size (mm)**					.34
	<53.8	114 (84.4)	210 (80.2)		
	≥53.8	21 (15.6)	52 (19.8)		
**Anteroposterior diameter of UF (mm)**					.11
	<52.7	83 (61.5)	138 (52.7)		
	≥52.7	52 (38.5)	124 (47.3)		
**Length of UF (mm)**					.60
	<60.1	75 (55.6)	137 (52.3)		
	≥60.1	60 (44.4)	125 (47.7)		
**Total energy (KJ)**					.29
	<398	91 (67.4)	191 (72.9)		
	≥398	44 (32.6)	71 (27.1)		
**Total irradiation time (s)**					.75
	<89.1	74 (54.8)	149 (56.9)		
	≥89.1	61 (45.2)	113 (43.1)		
**Total treatment time (m)**					.27
	<895.9	82 (60.7)	174 (66.4)		
	≥895.9	53 (39.3)	88 (33.6)		
**Position of UF**					.51
	Submucosal	5 (3.7)	17 (6.5)		
	Intramural	118 (87.4)	219 (83.6)		
	Subserosal	5 (3.7)	13 (5)		
**Number of UFs**					.44
	Single	47 (34.8)	89 (34)		
	Multiple	86 (63.7)	171 (65.3)		

^a^UF: uterine fibroid.

^b^LR: left to right.

#### Postoperative Factors

As shown in Table 6, the risk factor for postoperative lower abdominal pain was the length of fibroids, and the results were statistically significant (*P*=.03). The risk factor for menorrhagia in postoperative patients was the age at menarche, and the results were statistically significant (*P*=.02). The risk factor for postoperative menstrual blood clots was the number of fibroids, and the results were statistically significant (*P*=.04).

**Table 6:** Risk factors analysis of postoperative major symptomatic concepts.

Variables	Risk factor	Estimates (SE)	*P* value
Vaginal secretion	Menstrual period (days)	1.1421 (0.4358)	–.009
Dysmenorrhea disorder	None	—^a^	—
Menstrual disorder	None	—	—
Prolonged menstrual period	None	—	—
Lower abdominal pain	Length of UFs^b^	0.1190 (0.0563)	.03
Menorrhagia	Age of menophania	–1.0249 (0.4382)	.02
Menstrual blood clots	Number of UFs	2.3357 (1.1269)	.04
Dizziness	None	—	—
Fatigue	None	—	—

^a^Not applicable.

^b^UF: uterine fibroid.

## Discussion

### Principal Findings

For the first time, we extracted the concepts of patients’ symptoms from unstructured WeChat group chats text, demonstrating the feasibility of capturing what patients experienced after treatment from the most popular social media platform, WeChat, which features private communication between patients and medical staff. By combining medical records before surgery and WeChat communication after surgery, we profiled the patients’ experiences with UFs over the full course of surgical treatment. By establishing a relevant symptom corpus, we provided a research foundation to develop specific instruments for the extensive care of patients with UFs after surgery [6,19,20].

UFs are one of the most common benign tumors in women, although their prevalence may be underestimated in asymptomatic women. Approximately 25% to 50% of women with fibroids are symptomatic and experience heavy menses, reproductive issues, pain, increased urinary frequency, and anemia. Treatments for UFs, whether invasive, minimally invasive, or noninvasive, relieve disease-related symptoms and induce symptoms due to surgical insults [21]. Accurate monitoring of symptom trajectories over the course of surgery and recovery would help evaluate the treatment effect, manage postoperative complications, and alert about disease recurrence [22].

Using the real-world data from clinician-patient WeChat communication and the EHR, we identified 2 categories of symptoms, including disease-related symptoms and treatment-related symptoms. In this study, the top 5 symptoms collected during the postoperative rehabilitation of FUAS were vaginal secretion, dysmenorrhea, menstrual disorder, prolonged menstrual period, and lower abdominal pain. The symptoms involved in our study were similar to those in the study by Jeng et al [22], in which patients reported symptoms of UFs and health-related quality of life (Uterine Fibroid Symptom and Health-Related Quality of Life [UFS-QOL] questionnaire [23]). The application of a comprehensive questionnaire, such as the UFS-QOL, in clinical research, standardizes the outcome assessment but requires time and personnel resources, which may not be available in real-world practice for patient care. Moreover, although symptoms can be assessed using a disease-specific questionnaire, as a newly developed noninvasive surgical technique, FUAS-related symptoms have not been well validated in any PRO tool. Thus, the concept of symptoms in patients undergoing FUAS should first be based on qualitative studies.

Our study used NLP to identify symptoms experienced by patients after FUAS in WeChat group communication, providing a list of items that may serve as a draft of a FUAS-specific PRO symptom assessment tool. Although WeChat group communication may not produce a clear theme [24,25], it saves time and expenses for interviewer training, recruitment, and interviews required in a traditional qualitative study. Moreover, qualitative interviews are influenced by the current situation of patients [26], whereas the WeChat group communication provided the contemporary record of patients’ status and needs for health care during a period of time. In addition, compared with the small number of patients in a qualitative interview, the sample size of data from clinician-patient group communication could be large because of the popularity of WeChat in China. Thus, using WeChat data might improve the representativeness of patient experiences and could be representative of a more generalized population.

Social media communication may also inform patient management in the hospital [27,28]. Existing medical resources cannot fulfill patients’ needs on time, and it is also costly to consult outpatient services [29]. When minor problems emerge, people often turn to social media platforms to identify patients with similar experiences or web-based groups of doctors for advice. Reporting free texts is often unstructured but has potential. As patients actively report their health conditions, capturing adverse clinical events from this specific source is possible [30]. Conversely, medical staff can track patients’ health status and indicators on the web, collect relevant health data, and conduct appropriate scientific research activities.

One hurdle in using social media data is that text is unstructured. NLP technology has been described in the literature as an effective, inexpensive, and highly accurate tool for automatically identifying concepts from unstructured patient text [31,32]. NLP technology enables the accommodation of large unstructured data, significantly reducing time and labor costs that are spent in a traditional qualitative interview. It can quickly provide a framework for conventional qualitative analysis and then add patients in qualitative interviews to saturate it. Further research should be conducted on how NLP technology can be combined with qualitative interviews for the purpose of generating clinically meaningful results from unstructured medical documentations [24,25,33,34]

Our study achieved a high agreement between manual coding and Python-based text mining, similar to other studies that used SAS-based text mining [35-37]. This consistency suggests the potential of accurately extracting symptoms from free texts without the need for a lengthy, comprehensive manual review process. The process of NLP is not discourse or population specific, including compiling lists of search terms; manually reviewing samples of the data set to investigate abbreviations, misspellings, and negations; determining the order of search groups and elimination records; and investigating matches and mismatches throughout the process. This concept can be applied to other studies that investigate treatments in free texts.

### Limitations

First, the median period for data collection ended at 4-6 months after treatment, which is not sufficient for recurrence identification; this is a major concern in the management of UFs. Second, the symptoms identified from WeChat and the EHR were not scored for severity. Using the obtained list of symptoms, instruments with an appropriate scale for each symptom can be developed and validated for the precise evaluation of patients’ status. Third, the patients in this study were from a private hospital with a higher socioeconomic status than patients with UFs in the general population [1]. For broader interpretation, the current results should be validated in public hospitals in different areas.

### Conclusions

Our study showed that the extraction of a large amount of health data from social media is feasible. Python-based text mining is accurate and efficient in summarizing the symptoms experienced by patients with UFs under FUAS treatment. Mining algorithms provide a basis for the rapid and efficient analysis of large samples of free text in the future. The symptom burden of UFs treated with FUAS as summarized in this study can also provide a relevant reference for subsequent rehabilitation of patients.

## Appendix

Multimedia Appendix 1 Supplemental materials.

Multimedia Appendix 2 Distribution of the most common symptoms per category.

## Abbreviations

EHR: electronic health record
FUAS: focused ultrasound ablation surgery
GEE: generalized estimating equation
NLP: natural language processing
OR: odds ratio
PRO: patient-reported outcome
UF: uterine fibroid
UFS-QOL: Uterine Fibroid Symptom and Health-Related Quality of Life
