# Supplementary material for: Using Clinician-Patient WeChat Group Communication Data to Identify Symptom Burdens in Patients With Uterine Fibroids Under Focused Ultrasound Ablation Surgery Treatment: Qualitative Study
Source: JMIR Form Res. 2023 Sep 1;7:e43995. doi: 10.2196/43995 (PMC10504630; doi:10.2196/43995)

**Appendix List of Contents**

Figure S1. Doctor-patient group screenshot data display

Figure S2. Frequency distribution of manually coded symptoms using Doccano

Figure S3. Text string search order for Python-based text mining of uterine fibroids WeChat group-chats

Table S1. Manual annotation of a corpus of major symptoms

Table S2. Corpus of symptoms in patients with uterine fibroids after HIFU surgery/mapping of symptoms to canonical forms (English)

Table S3. Corpus of symptoms in patients with uterine fibroids after HIFU surgery/mapping of symptoms to canonical forms (Chinese)

Table S1. Manual annotation of a corpus of major symptoms

| variables | annotation records | Regular expression algorithm |
| --- | --- | --- |
| dymenorrhea | 经前和经结束时后背下腰处痛经严重/Severe menstrual pain in the lower back before and at the end of the period | 痛经/dysmenorrhea |
|  | 手术后月经第二个周期月经量减少但做手术的右下腹疼痛如痛经般疼痛这在术前是未有的/The second cycle of menstruation after the surgery was less frequent, but the pain in the right lower abdomen was like menstrual cramps, which was not present before the surgery. | 痛经/dysmenorrhea |
| menorrhagia | 月经量大/Heavy menstrual flow | 量大/heavy flow |
|  | 就是做完第二个月来例假量特别大/It is the second month after the period is particularly large volume | 例假、量特别大/Periods, unusually high volume |
| vaginal discharge | 我做了手术了天了第五天开始有点东西流出来但是这两天流血比较多我月经量本身就很多所以没超过经量我不晓得这是不是/I've had the surgery for a few days now and on the fifth day something started to flow out but the bleeding has been more in the past two days I have a lot of menstrual flow so I don't exceed the amount of menstruation I don't know if this is? | 东西流出/Something flows out |
|  | 我这两天流出来比较多呢还有红色液体流出来量不多但是一天都要垫卫生棉一天要流出来好几次这样有撒子没得/I have been flowing out more in the past two days, but the amount of red liquid flowing out is not much, but a day to pad tampons a day to flow out several times so that there is no sazi. | 红色液体/Red liquid |
| menstrual disorder | 我是号做的手术来的第一次月经号来第二次月经提前的一个星期以前的月经是准时天这正常吗/I had my first period on the first day of surgery, and my second period was a week early, and my previous period was on time. | 月经提前/advanced menstrual period |
|  | 想咨询一下做完手术之后一直没来月经会不会绝经啦/I would like to ask if my menstruation will be menopause if I haven't been menstruating since the operation | 一直没来月经/haven't had my period |
| menstrual blood clots | 术后运动第三天打卡头两天排除的是粉色的水今天排除绿豆大小粉红色血块少量出血其他一切还好/On the third day of exercise after surgery, pink water was excluded in the first two days of clocking. Today, green bean sized pink blood clots were excluded, and a small amount of bleeding occurred. Everything else was OK | 血块/menstrual blood clots |
|  | 第一天血的颜色比较深肚子疼喝了红糖水以后血量大了排的血块多了/The color of the blood on the first day was quite dark. My stomach ached. After drinking brown sugar water, my blood clots were large. I discharged more blood clots | 血块/blood clots |
| Lower abdominal pain | 我这几天一直感觉肚子隐痛/I've been feeling a dull pain in my stomach these days | 肚子隐痛/Cryptic abdominal pain |
|  | 早上肚子到会阴部抽了几下，现在肚子痛得很，肚子有点胀胀的总体感觉/In the morning, I had a few abdominal cramps in the perineum. Now my stomach is very painful, and I feel a little bloated | 肚子痛/upset stomach |
| prolonged menstrual period | 我是四月九号做的手术，月经是四月二十八号来的到今天都十天了还没干净/I had an operation on April 9. My menstruation came on April 28. It has been ten days since today | 还没干净/Not clean yet |
|  | 我做过海扶手术个月了这次月经一直不断有半个月了见的也不多就暗红色的有事吗用治疗不用/I've had a HIFU operation for months. This period has been going on for half a month. I haven't seen much, but it's dark red. What's the matter? I don't need treatment | 月经一直不断/Menstruation keeps going |
| mood | 实在不行可以看看医生不要太焦虑了/I really can't see the doctor. Don't be too anxious | 焦虑/anxious |
|  | 思想放松,紧张焦虑情绪也有影响/Relaxed thoughts, tension and anxiety also affect | 焦虑/anxiety |
| dizzy | 伤口估计正常的有点痒其它的都还可以精神也算好已经开始上半天班了久了有点晕感觉是一天比一天好/The wound is supposed to be normal, a little itchy, and the rest of us are in good spirits. We have been working half a day for a long time. I feel a little dizzy. It's getting better day by day | 晕/dizzy |
|  | 例假来得多头晕再抽血检查激素可以吗/Is it OK to draw blood to check hormone after more dizziness during the regular holidays | 头晕/dizziness |
| fatigue | 感觉很累/I feel very tired | 累/tired |
|  | 最近身体好疲惫/I'm tired recently | 疲惫/tired |

Figure S1. Doctor-patient group screenshot data display


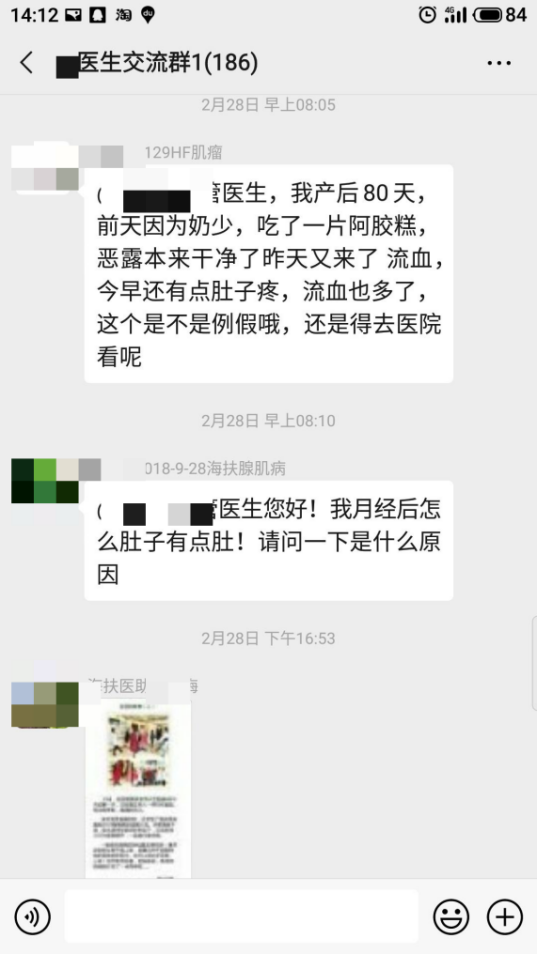


Table S2. Corpus of symptoms in patients with uterine fibroids after HIFU surgery/mapping of symptoms to canonical forms (English)

| Normalized  symptom | Symptom variants |
| --- | --- |
| Dysmenorrhea | Pressing pain, throbbing pain, touching pain, bouncing pain, abdominal wall pain, groin pain, dragging pain ,menstrual pain, mild pain, falling pain ,general pain, concealed pain, Yin pain, swell pain s,welling pain ,labor burning pain, stabbing pain, tingling pain, regular pain |
| Lower abdominal pain | pain in the abdomen, pain in the lower abdomen ,pain in the groin ,pain in the abdominal wall,abdominal pressing pain ,abdominal throbbing pain, abdominal touching pain,abdominal bouncing pain, abdominal wall pain ,abdominal groin pain, abdominal dragging pain, abdominal menstrual pain, abdominal mild pain, abdominal falling pain, abdominal general pain,abdominal concealed pain ,abdominal Yin pain ,abdominal swell pain ,abdominal labor burning pain, abdominal stabbing pain ,abdominal tingling pain,abdominal regular pain |
| Menorrhagia | Quite a lot of menstruation, a lot of menstruation, a little more menstruation, a good deal of menstruation,a great many of menstruation |
| Abdominal discomfort | Straining,tenesmus,meteorism, tympanites, ballooning, tympania, tympanism, gelosis, feel unwell , feel ill , suffer pain,aching and limp, move up and down ,swell,falling |
| Vaginal secretion | Increased secretions, watery secretions ,bloody secretions, excreted secretions ,a little secretions ,a lot of secretions,Black discharge, brown discharge, brown discharge ,light red discharge, pink discharge ,dark red discharge |
| Menstrual disorder- | Irregular menstruation, early menstruation, delayed menstruation, menstruation comes again, menstruation is not very normal, menstruation is a little chaotic, delayed menstruation, menstrual leave has not come, menstrual leave is not normal, menstrual leave is early, menstrual leave is delayed, menstrual leave is irregular, two months have not come to menstrual leave, big aunt is delayed, big aunt is advanced, big aunt is delayed |
| Menstrual blood clots | Blood clot, small blood clot, large blood clot, a lot of blood clots, a lot of blood drops quickly, a little blood clot, all blood clots, a lot of blood clots, so many blood clots, big lump,many blood clots, pink blood clots, black blood clots, brown blood clots , coagulum , grume , clot,curd |
| prolonged menstrual period | Menstruation has been unclean, menstruation is not clean, menstruation has been continuous, menstrual period has been prolonged, menstruation has not stopped for half a month, menstruation time is long, menstruation has been lasted for Days, menstruation does not mean to go, Menstruation takes half a month to complete. There are many menstrual periods and endless menstrual holidays |
| Mood | be fidgety, be agitated , irritable, be in a fret,feel anxious ,extremely anxious , have worries and , misgivings , inquietude, In the Mood , Feel Good,Vexed, worried, distressed , tormente |
| Lower body discomfort | Swollen and sour feet, uncomfortable numbness, fever in the center of the feet, numbness in the legs and feet, numbness in the ends of the hands and feet, acid swelling in the knee joints, soft swelling in the legs, soft swelling in the legs, acid swelling in the legs |
| Waist discomfort | The waist is a little swollen, the waist is sour, the waist will be sour, the waist is sleepy, the waist is uncomfortable, the waist and abdomen are weak, the waist is a little swollen, and the waist is very swollen |
| Dizzy | a little dizzy,dizzy frequently,dizzy occurs,feel dizzy and with one's eyesight dimmed, feel faint and vision blurred; groggy, mentally confused, giddy,One's head began to swim and one's eyes were misted, One's wits are wandering,slaphappy, with head giddy and eyes dazzled |
| Leucorrhea abnormality | There are many leucorrhea, leucorrhea is red, leucorrhea is yellow, leucorrhea is bloody, leucorrhea is brown, leucorrhea has blood, leucorrhea secretion is more, leucorrhea is thin, leucorrhea quantity is more, leucorrhea is light yellow, leucorrhea is quite many, leucorrhea is brown |
| Fatigue | Exhausted (a little, very, extremely) tired and flustered, tired and weak,Unable to apply force, have no strength,fatigue |
| Sleeping barrier | Insomnia,can't sleep well,sleep quality is poor sleep, can't sleep normally ,sleep is not relieved , sleep is not very good ,sleep is particularly bad |
| Abnormal urination | Uncomfortable urination, frequent urination, urgent urination, bloody urination, increased urination, uncontrolled urination |
| Itch | It's a little itchy in the vulva. It's itchy in the vulva. It's itchy in the belly. It's itchy in the lower body |
| Nausea-vomiting | Nausea and vomiting, have nausea symptoms, nausea and vomiting, continuous vomiting |
| [abnormal](javascript:;) [defecation](javascript:;) | Poor defecation, The operation has not defecated until now, The stool can't be solved, I haven't defecated all the time,stool three or four times a day |
| Perianal discomfort | Anal pressure ,anal pendant, anal distension ,anal distension ,anal perianal pendant distension, anal pendant |

Table S3. Corpus of symptoms in patients with uterine fibroids after HIFU surgery/mapping of symptoms to canonical forms(Chinese)

| 痛经-227 | 按压痛 抽痛 触摸痛 弹跳痛 腹壁痛 腹股沟痛 绞痛 牵扯痛 酸痛 痛经痛 微痛 坠痛 一般痛 隐痛 阴痛 胀痛 涨痛 阵痛 灼痛 刺痛 麻痛 常规痛 痛 |
| --- | --- |
| 腹痛-123 | 肚子痛 腹部痛 腹部疼 下腹 肚子疼 腹疼 腹股沟痛 腹壁痛  按压痛 抽痛 触摸痛 弹跳痛 腹壁痛 腹股沟痛 绞痛 牵扯痛 酸痛 痛经痛 微痛 坠痛 一般痛 隐痛 阴痛 胀痛 涨痛 阵痛 灼痛 刺痛 麻痛 常规痛 痛 |
| 月经量多，增多-76 | 月经挺多 月经量多 月经量大 月经量比以前多 月经量特别大 月经量很多 月经量有点多 月经量稍多 月经量超多 月经量较大 例假很多 例假血量大  例假量多 大姨妈好多 大姨妈量多 |
| 腹部不适-70 | 坠胀 下坠 坠涨 鼓 鼓胀 变大 腹部硬块 不舒服 难受 酸软 跳动 肿 有点涨 发胀 涨的很 |
| 阴道分泌物-61 | 分泌物增多 水状分泌物 分泌物带血 有分泌物 排分泌物 出现分泌物 有点分泌物 分泌物很多  黑色分泌物 褐色分泌物 咖啡色分泌物 淡红色分泌物 粉色分泌物 暗红色分泌物 |
| 月经紊乱-60 | 月经乱 月经提前 月经推迟 月经推后 月经又来 月经不是很正常 月经有点乱 月经延迟 例假一直没来 例假不太正常 例假提前 例假推迟 例假不规律  两个月没来例假 大姨妈推迟 大姨妈提前 大姨妈延迟 |
| 经血成块-60 | 血块 血凝块 小血块 大血块 大量血块 血块多 大量掉血快 一点血块 全是血块 有点血块 很多血块 好多血块 这么多血凝块 大坨大坨血块 血块多  粉红色血块 黑色血块 褐色血块 |
| 月经经期延长-30 | 月经一直不干净 月经不干净 月经一直不断 经期延长 月经半个月没停 月经时间长 月经都.....天了 月经没有走的意思 月经半个月才完事 经期多  例假淋漓不尽 |
| 心情干扰-22 | 烦躁 焦虑 情绪不好 心情不好 苦恼 |
| 下肢不适-21 | 脚肿 酸 不舒服 麻 脚心发热 腿脚发麻 麻木感 手脚末端发麻 膝盖关节酸胀感 腿软 腿软胀 小腿酸 |
| 腰不适-20 | 腰部有点胀 腰部酸胀 腰会酸 腰困 腰部不适 腰酸 腰不舒服 腰腹无力 腰有点涨 腰很胀 |
| Dizzy-20 | 有点晕 头昏眼花 头晕 晕趴 经常头晕 发晕 头晕脚轻 出现头晕 |
| 白带异常-17 | 白带很多 白带带红 白带带黄 白带带血丝 白带褐色 白带有血 白带分泌物较多 白带稀 白带量多 白带淡黄色 白带挺多 白带咖啡色 |
| Fatigue-16 | 体力不支 （有点、非常 很）累 累得慌 疲惫 困 乏力 没劲 使不上力 浑身没力气 无力 |
| 睡眠障碍-14 | 失眠 睡不着觉 睡眠不好 睡不着 睡眠质量不好 睡眠很差 无法正常睡眠 睡眠没缓解 睡不好 睡眠不太好 睡的特别差劲 |
| 小便不适-13 | 小便难受 尿频 尿急 小便带血 小便增多 小便不受控制 |
| 痒-10 | 外阴有点痒 外阴好痒 外阴有一点痒 肚子有点痒 下身有点痒 |
| 恶心呕吐-9 | 恶心 呕吐 有恶心症状 恶心想吐 持续吐 |
| 大便不适-7 | 排便不好 手术到现在还未排便 大便解不出 一直都没排便 大便一天三四次 |
| 肛周不适-6 | 肛门压迫感 肛门坠胀 肛门胀 肛周坠胀 肛门坠涨 |

Figure S2. Frequency distribution of manually coded symptoms using Doccano


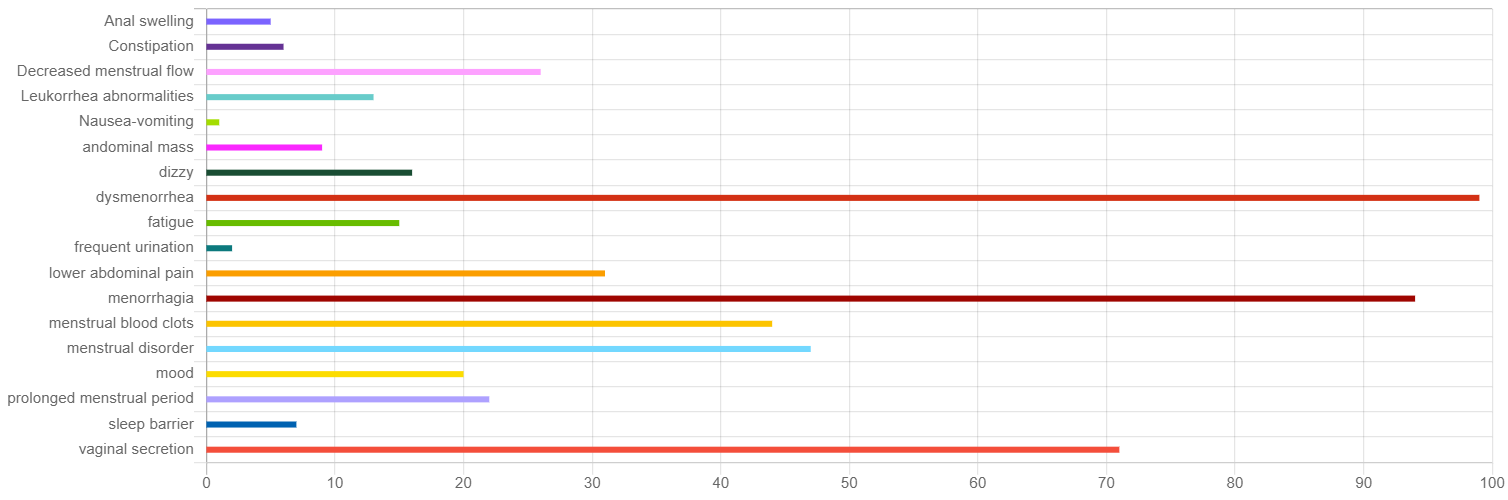


Figure S3. Text string search order for Python-based text mining of uterine fibroids WeChat group-chats


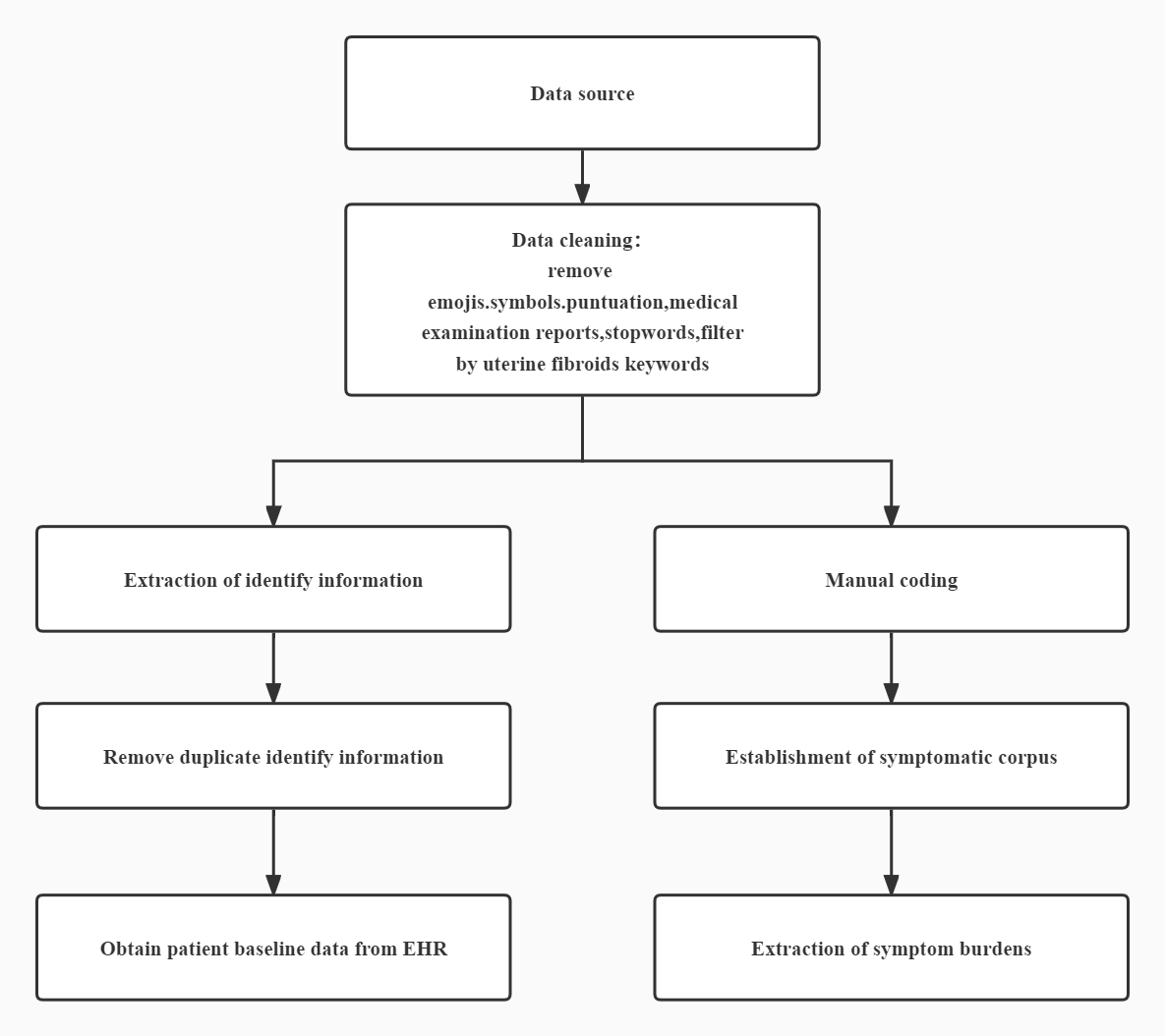

Supplement: Multimedia Appendix 1 [file formative_v7i1e43995_app1.docx]
