# Supplementary material for: Using Clinician-Patient WeChat Group Communication Data to Identify Symptom Burdens in Patients With Uterine Fibroids Under Focused Ultrasound Ablation Surgery Treatment: Qualitative Study
Source: JMIR Form Res. 2023 Sep 1;7:e43995. doi: 10.2196/43995 (PMC10504630; doi:10.2196/43995)
Supplement: Multimedia Appendix 2 [file formative_v7i1e43995_app2.docx]

Multimedia Appendix 2. Distribution of the most common symptoms per category

| Distribution of preoperative symptoms | Distribution of postoperative symptoms |
| --- | --- |
|  |  |
| 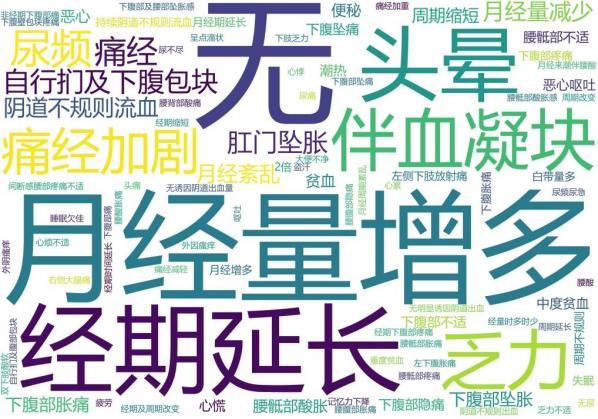 | 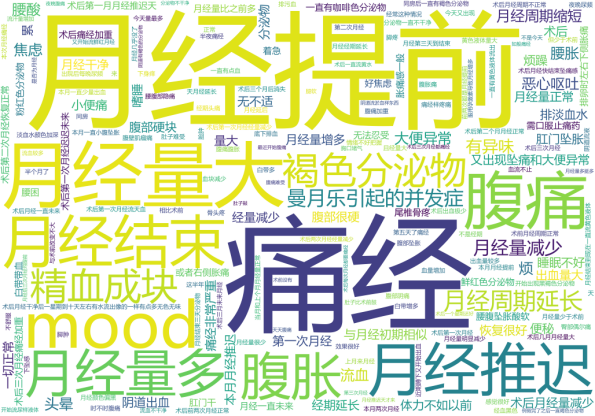 |
| \| Present symptoms \| Frequency \| \| --- \| --- \| \| Menorrhagia \| 137 \| \| Dysmenorrhea \| 135 \| \| Prolonged menstrual period \| 86 \| \| Dizziness \| 56 \| \| Fatigue \| 56 \| \| Menstrual blood clots \| 56 \| \| Lower abdominal pain \| 44 \| \| Intensified dysmenorrhea \| 40 \| \| Frequent urination \| 26 \| \| Abdominal mass \| 15 \| \| Secondary dysmenorrhea \| 15 \| \| Irregular vaginal bleeding \| 15 \| \| Anal distension \| 11 \| \| Menstrual period disorder \| 9 \| \| Menstrual cycle disorder \| 8 \| \| Constipation \| 4 \| \| Palpitation \| 4 \| \| Nausea and vomiting \| 3 \| \| Hot flashes \| 3 \| \| Increased leucorrhea \| 3 \| \| Night sweat \| 2 \| \| Insomnia \| 2 \| \| Headache \| 2 \| | \| Present symptoms \| Frequency \| \| --- \| --- \| \| Vaginal secretion \| 64 \| \| Dysmenorrhea \| 64 \| \| Menstrual disorder \| 62 \| \| Secondary dysmenorrhea \| 38 \| \| Prolonged menstrual period \| 34 \| \| Lower abdominal pain \| 33 \| \| Menstrual blood clots \| 28 \| \| Mood \| 26 \| \| Menorrhagia \| 17 \| \| Dizziness \| 10 \| \| Anal distension \| 5 \| \| Frequent urination \| 4 \| \| Abdominal mass \| 3 \| \| Nausea \| 3 \| \| Vomiting \| 3 \| \| Fatigue \| 3 \| \| Abnormal leucorrhea \| 3 \| \| Constipation \| 2 \| \| Palpitation \| 1 \| \| Hot flashes \| 1 \| \| Night sweats \| 1 \| \| Insomnia \| 1 \| \| Headache \| 1 \| |
